# Changes of Metabolic Biomarker Levels upon One-Year Anti-TNF-α Therapy in Rheumatoid Arthritis and Ankylosing Spondylitis: Associations with Vascular Pathophysiology

**DOI:** 10.3390/biom11101535

**Published:** 2021-10-18

**Authors:** Monika Czókolyová, Anita Pusztai, Edit Végh, Ágnes Horváth, Anita Szentpéteri, Attila Hamar, Szilvia Szamosi, Katalin Hodosi, Andrea Domján, Sándor Szántó, György Kerekes, Ildikó Seres, Mariann Harangi, György Paragh, Éva Szekanecz, Zoltán Szekanecz, Gabriella Szűcs

**Affiliations:** 1 Division of Rheumatology, Faculty of Medicine, University of Debrecen, 4031 Debrecen, Hungary; monika.czokolyova@gmail.com (M.C.); anita.pusztai01@gmail.com (A.P.); veghe22@gmail.com (E.V.); kis.horvathagi@gmail.com (Á.H.); attilahamar.2010@gmail.com (A.H.); szamosi.szilvi@gmail.com (S.S.); khodosi@gmail.com (K.H.); domjan.andrea@gmail.com (A.D.); szanto.sandor@med.unideb.hu (S.S.); szucs.gabriella@med.unideb.hu (G.S.); 2 Division of Metabolic Diseases, Department of Medicine, Faculty of Medicine, University of Debrecen, 4031 Debrecen, Hungary; szentpeteri.anita@gmail.com (A.S.); seres@internal.med.unideb.hu (I.S.); harangi@belklinika.com (M.H.); paragh.gyorgy@med.unideb.hu (G.P.); 3 Department of Sports Medicine, Faculty of Medicine, University of Debrecen, 4031 Debrecen, Hungary; 4 Intensive Care Unit, Department of Medicine, Faculty of Medicine, University of Debrecen, 4031 Debrecen, Hungary; gkerekesg@gmail.com; 5 Department of Oncology, Faculty of Medicine, University of Debrecen, 4031 Debrecen, Hungary; szevadr17@gmail.com

**Keywords:** rheumatoid arthritis, ankylosing spondylitis, biologic therapy, metabolic biomarkers, lipids, adipokines

## Abstract

Background: Cardiovascular (CV) morbidity, mortality, and metabolic syndrome are associated with rheumatoid arthritis (RA) and ankylosing spondylitis (AS). Here, lipids and other metabolic markers in relation to vascular function and clinical markers were evaluated in RA and AS patients undergoing one-year anti-TNF therapy. Patients and methods: Fifty-three patients including 36 RA patients treated with either etanercept (ETN) or certolizumab pegol (CZP) and 17 AS patients treated with ETN were included in a 12-month follow-up study. Various lipids, paraoxonase (PON) and arylesterase (ARE) activities, myeloperoxidase (MPO) and adipokine levels were determined overtime. Ultrasonography was performed to determine flow-mediated vasodilation (FMD), common carotid intima-media thickness (ccIMT), and arterial pulse-wave velocity (PWV) in all patients. All assessments were performed at baseline and 6 and 12 months after treatment initiation. Results: Anti-TNF therapy decreased ARE activity, MPO, adiponectin, and chemerin levels after 12 months (*p* < 0.05). Lipids, PON activity, and leptin remained unchanged. Regression analyses suggested variable associations of IMT, PWV, and FMD with ARE, MPO, leptin, and lipids (*p* < 0.05). On the other hand, these metabolic parameters were significantly associated with disease duration, CV history, CRP, obesity, PWV, and IMT (*p* < 0.05). One-year anti-TNF treatment together with baseline leptin (*p* = 0.039) or CRP (*p* = 0.016) levels determined 12 months of lipid changes overtime. TNF inhibition together with baseline disease activity determined ARE activity changes (*p* = 0.046). Anti-TNF therapy and baseline chemerin levels determined IMT changes overtime (*p* = 0.003). Conclusions: Assessment of various metabolic parameters together with disease activity, CRP, and ultrasound-based techniques may exert additional value in determining CV burden and in monitoring the effects of biologics on preclinical vascular pathophysiology.

## 1. Introduction

Arthritides, such as rheumatoid arthritis (RA) and ankylosing spondylitis (AS), have been associated with accelerated, inflammatory atherosclerosis, increased cardiovascular (CV) morbidity and mortality, as well as metabolic changes [1,2,3,4,5,6,7,8,9]. Systemic inflammation and inflammatory mediators are crucial in the premature atherosclerotic events in rheumatic conditions [3,8,9,10,11]. It is necessary to detect CV abnormalities early, possibly in the preclinical phase of CV disease (CVD) [1]. Moreover, targeted therapies, such as tumor necrosis factor α (TNF-α) inhibitors may dampen secondary inflammatory atherosclerosis and may prevent major CV events (MACE), particularly in anti-TNF-responders [1,12,13,14,15].

Increased risk of metabolic syndrome has been associated with arthritis [5,6,7,8,9]. Several metabolic factors are involved in the development of atherosclerosis in the general population, as well as in patients with inflammatory rheumatic musculoskeletal diseases (RMD). With respect to lipids, systemic inflammation associated with RA and AS may result in decreased lipid levels hence the terminology “lipid paradox” [5,16,17,18]. Biologics, primarily IL-6, and JAK inhibitors, but, to a lesser extent, also TNF-α blockers may decrease inflammation and C-reactive protein (CRP) and, inversely, increase the levels of total cholesterol (TC), low-density lipoprotein (LDL-C), high-density lipoprotein cholesterol (HDL-C), and triglycerides (TG) [5,6,16,17,19,20]. However, the so-called atherogenic index (TC/HDL-C or LDL-C/HDL-C) remained unchanged in most studies suggesting that changes in lipid levels may not confer increased CV risk [5,6,16,17,18,19,20]. Thus, lipid elevation upon targeted therapies may reflect the favorable effect of these agents on systemic inflammation [5,14,16,17].

Serum paraoxonase/arylesterase 1 (PON1/ARE), encoded by the *PON1* gene in humans, is an HDL-associated hydrolase carried on apoA-I apoprotein, which inhibits low-density lipoprotein oxidation. PON1 is an antioxidant that reduces LDL oxidation and prevent atherosclerosis [21,22]. Apart from its PON activity, PON1 also exerts ARE activity [22]. Impaired PON1 PON and ARE activity has been found in inflammatory diseases associated with accelerated atherosclerosis, such as RA [23,24] and AS [25,26]. PON1 is not only associated with CVD in arthritis but may also dampen inflammation as shown in PON1-transgenic mice [27]. In RA, there was an inverse correlation between disease activity and PON1 [28]. In one study, serum PON and ARE activities correlated with rheumatoid factor (RF) and anti-citrullinated protein antibody (ACPA) levels in RA [29]. We have found correlation between PON activity and serum TNF-α levels in RA suggesting that PON1 production may be a result of a feedback response to cytokine release [11]. There have been very few studies on the effects of biologics on PON1 PON and ARE activities. In one study, six-month anti-TNF treatment increased PON activity in RA [30]. In psoriasis, etanercept increased PON activity [31]. There have been no reports on the relationship of ARE and biologics.

Myeloperoxidase (MPO), encoded by the *MPO* gene in humans, is a peroxidase most abundantly found in neutrophils. MPO is involved in neutrophil oxidative burst and has been associated with atherosclerosis, the development of unstable plaques and CVD [32,33,34]. We have found negative correlation between MPO and PON activity in the general population [34]. There have been relatively few studies on the role of MPO in RA. There are increased plasma MPO levels in RA [35,36] and MPO may have a role in the generation of citrulline and homocitrulline in the synovium [37]. MPO is also involved in RA-related oxidative stress [36]. Only 12% of RA patients without evidence of vasculitis had anti-MPO antibodies [38]. Very little information is available on the role of MPO in AS or on the possible effects of biologics on MPO production in RMDs.

Adipokines are protein mediators released by adipose tissue involved in the pathogenesis of atherosclerosis and inflammation [5,39]. Adipokines may exert pro- or anti-atherogenic and also pro- or anti-inflammatory features. Among others, adiponectin, leptin, and chemerin have been associated with arthritis and also with RMD-associated CVD [5,6,39]. In general, adiponectin and PON exert a positive association [40]. Leptin is a pro-inflammatory adipokine that controls body weight and appetite, and it is a major determinant of obesity [5,6,39]. Leptin reduces PON activity and aggravates insulin resistance [39,41]. Chemerin is a chemoattractant adipokine expressed in immune cells, as well as fat tissue. Chemerin triggers various pro-inflammatory processes in arthritides in part by the activation of synovial fibroblasts [5,39,42]. Chemerin has also been implicated in arthritis-related CVD [43]. Biologics, such as TNF inhibitors have differential effects on adipokine production [5]. In some studies, biologics suppressed chemerin levels in arthritis [5,44,45]. On the other hand, there have been lots of controversy with respect to the effects of anti-TNF agents on circulating levels of adiponectin and leptin [5,6,14,39,46].

Vascular pathophysiology might be investigated by various ultrasound-based techniques. Common carotid intima-media thickness (IMT), brachial artery flow-mediated vasodilation (FMD), and arterial pulse-wave velocity (PWV) are suitable to detect overt atherosclerosis, endothelial dysfunction, and vascular stiffness, respectively [4]. Increased IMT and PWV, as well as impaired FMD have been associated with RA and AS [4,11,47,48,49]. IMT may show correlation with PON activity [50]. Anti-TNF treatment may, at least transiently, improve endothelial function, atherosclerosis, and arterial stiffness (reviewed in [14]).

There have been very few studies on the role of PON/ARE and MPO in CV comorbidities underlying inflammatory rheumatic diseases. In addition, little information has become available how targeted therapies influence these metabolic pathways. Therefore, in this mixed cohort of RA and AS patients, for the first time, we wished to study the effects of one-year TNF inhibition on PON/ARE, MPO the above discussed metabolic markers in association with disease activity, lipids, adipokines, namely, leptin, adiponectin, and chemerin, and vascular pathophysiology including IMT, FMD, and PWV. We have not found similarly complex study in the literature. In the same cohort, we have assessed changes of IMT, FMD, and PWV upon anti-TNF therapy. Significant improvement of FMD and PWV, as well as halted progression of IMT were observed [47]. Now our aim was to determine the follow-up effects of anti-TNF therapy on metabolic biomarkers including PON/ARE, MPO, lipids, and adipokines and to link this to various immune-inflammatory effects, as well as vascular pathophysiology in RA and AS.

## 2. Patients and Methods

### 2.1. Patients

Fifty-three consecutive patients with inflammatory arthritis (36 RA and 17 AS) who required biological therapy but who were unselected for CVD were enrolled in the study. Exclusion criteria included untreated hypertension (blood pressure > 140/90 mmHg), congestive heart failure, current inflammatory disease other than RA or AS, infectious disease or renal failure (serum creatinine ≥ 117 mmol/L). None of patients received aspirin, clopidogrel, heparin, or warfarin at the time of inclusion. Certainly hypertension was controlled by antihypertensive drugs. Patients with active disease were recruited prior to initiating a biological therapy. All patients started on an anti-TNF therapy at baseline and received the same biological treatment at one year. Among the 36 RA patients, 20 received etanercept (ETN) 50 mg/week subcutaneous (SC) and 16 received certolizumab pegol (CZP) (400 mg at 0, 2, and 4 weeks, and thereafter 200 mg twice weekly SC). Altogether 28 RA patients received methotrexate (MTX) in combination with the anti-TNF treatment. These patients had been on MTX prior to the initiation of biologics and the MTX dose was not changed. All 17 AS patients received ETN 50 mg/week SC. Although most RA patients and some AS patients may have received corticosteroids prior to the study, none of the patients were on corticosteroids for at least 3 months prior to and during the study. Patients did not receive any conventional DMARDs other than MTX and they only took non-steroidal anti-inflammatory drugs on demand. Some patients had been receiving statins for at least 3 months prior to the study and the dose remained unchanged during the study.

Disease activity was determined by DAS28 [51] and BASDAI [52] in RA and AS, respectively.

The study was approved by the Hungarian Scientific Research Council Ethical Committee (approval No. 14804-2/2011/EKU). Written informed consent was obtained from each patient and assessments were carried out according to the Declaration of Helsinki.

### 2.2. Clinical Assessment

First, detailed medical history was taken. We inquired for history of CVD, obesity, diabetes, hypertension (treated), as well as current smoking during the last 2 years prior to the start of this study by a questionnaire (Table 1). Further clinical assessments including physical examination were performed at baseline and after 3, 6, and 12 months of therapy.

### 2.3. Laboratory Measurements

Venous blood samples were taken after an overnight fast and sera were prepared immediately. Lipid analyses including TC, LDL-C, HDL-C, TG, and lipoprotein (a) [Lp(a)] were performed from fresh sera with Cobas c501 autoanalyzer (Roche Ltd., Mannheim, Germany). Full blood count including hemoglobin (hgb), hematocrit (htc), white blood cells (WBC), red blood cells (RBC), platelets (PLT), as well as neutrophil and lymphocyte absolute counts were determined by routine laboratory analyses at all time points.

PON1 PON activity (U/L) was determined on a microtiter plate by a kinetic, semi-automated method using paraoxon (O,O-diethyl-O-p-nitrophenyl-phosphate, Sigma Aldrich) as a substrate [53,54]. PON1 ARE activity (U/L) was assayed with a phenylacetate substrate (Sigma Aldrich Brand, Merck, Darmstadt, Germany) and the hydrolysis of phenylacetate was monitored at 270 nm [34,53]. Serum MPO concentrations (ng/mL) were determined by a commercially available ELISA kits (R&D Systems, Minneapolis, MN, USA) with 6.6–7.7 CV% intra-, and 6.5–9.4 CV% inter-assay variabilities [34]. Among adipokines, serum chemerin (ng/mL), leptin (ng/mL) and adiponectin concentrations (μg/mL) were determined by a commercially available ELISA kits (R&D Systems, Minneapolis, MN, USA) [53]. Leptin/adiponectin ratio was also calculated.

Serum high sensitivity C reactive protein (hsCRP) and IgM rheumatoid factor (RF) were measured by quantitative nephelometry (Cobas Mira Plus-Roche), using CRP and RF reagents (both Dialab). ACPA (anti-CCP) autoantibodies were detected in serum samples using a second generation Immunoscan-RA CCP2 ELISA test (Euro Diagnostica, Malmö, Sweden) [47]. The assay was performed according to the manufacturer’s instructions.

### 2.4. Assessment of Vascular Physiology by Ultrasound

The FMD, IMT, and PWV assessments carried out as analyzed and reported previously [47]. Here, we used those results in order to correlate metabolic data with vascular pathophysiology.

### 2.5. Statistical Analysis

Statistical analysis was performed using the SPSS version 22.0 (IBM) software. Data are expressed as the mean ± SD for continuous variables and percentages for categorical variables. Continuous variables were evaluated by paired two-tailed *t*-test and Wilcoxon test. Nominal variables were compared between groups using the chi-squared or Fisher’s exact test, as appropriate. Correlations were determined by Pearson’s and Spearman’s analyses. Univariate and multiple regression analysis using the stepwise method was applied to investigate independent associations between angiogenic biomarkers (dependent variables) and other clinical, laboratory and imaging parameters (independent variables). The β standardized linear coefficients showing linear correlations between two parameters were determined. The B (+95% CI) regression coefficient indicated independent associations between dependent and independent variables during changes. Repeated measures analysis of variance (RM-ANOVA) was performed in order to determine the additional effects of multiple parameters on changes of vascular imaging markers between baseline and 12 months. The dependent variables were the metabolic biomarkers. RM-ANOVA uses the F-test resulting in the F values along with the p values. Partial η^2^ is given as indicator of effect size ranging from 0 to 1, with values of 0.01 suggesting small, 0.06 medium, and 0.14 large effects.

Sample size analysis was performed by the GPower 3.1.9.2 software. Baseline and 12-month ARE, MPO, and chemerin level changes were considered. Considering 80% power, statistically significant changes are observed using minimum patient numbers of 32, 12, and 16, respectively.

Regarding reproducibility of the vascular ultrasound assessments, all measurements were performed by a single observer (G.K.). Intraobserver variability of FMD, IMT, and PWV measurements were calculated as 5%, 4.2%, and 3.3%, respectively. The “stability” of measurements is indicated by the reproducibility for month-to-month repeated assessments of these parameters. According to the Brand–Altman analysis, the 95% limits of agreement ranged between −1.6% and 1.9% for all assessments. For example, with respect to 0-6-12-month changes of FMD, the intra-class correlation coefficient was 0.633 (*p* < 0.001) [11]. In all analyses, *p*-values < 0.05 were considered significant.

## 3. Results

### 3.1. Patient Demographics

Patient characteristics are seen in Table 1. The cohort included 34 women and 19 men with mean age of 52.0 ± 12.1 (range: 24–83) years. Mean disease duration was 8.5 ± 7.9 (range: 1–44) years, while mean age at diagnosis was 43.5 ± 12.1 (range: 23–62) years. At baseline RA patients had a mean DAS28 of 5.00 ± 0.86, while AS patients exerted mean BASDAI of 5.79 ± 1.19 (Table 1).

### 3.2. Effects of TNF Inhibition on Circulating Metabolic Biomarkers

**Figure 1:**
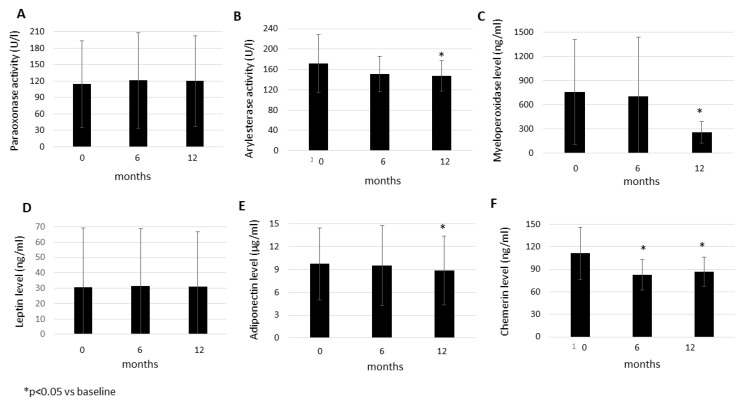
Effects of one-year anti-TNF therapy on paraoxonase activity (**A**), arylesterase activity (**B**), as well as myeloperoxidase (**C**), leptin **(D**), adiponectin (**E**), and chemerin (**F**) levels in RA and AS. Asterisks indicate significant changes (*p* < 0.05).

In the mixed cohort of 53 RA and AS patients, PON activity only numerically increased after 6 months (121.0 ± 87.4 U/L; *p* = 0.107) and 12 months (120.1 ± 82.6 U/L; *p* = 0.140) compared to baseline (114.1 ± 79.3 U/L) (Figure 1A). PON1 ARE activity numerically decreased after 6 months (150.9 ± 34.7 U/L; *p* = 0.052) and significantly after 12 months (147.4 ± 29.5 U/L; *p* = 0.027) compared to baseline (171.4 ± 56.6 U/L) (Figure 1B). MPO also numerically decreased after 6 months (703.0 ± 738.1 ng/mL; *p* = 0.097) and significantly after 12 months (255.5 ± 137.3 ng/mL) versus baseline (758.6 ± 651.4 ng/mL; *p* < 0.001) (Figure 1C).

Among adipokines, leptin levels did not change after 6 (31.4 ± 37.4 ng/mL; *p* = 0.642) or 12 months (31.0 ± 35.9 ng/mL; *p* = 0.918) versus baseline (30.6 ± 38.7 ng/mL) (Figure 1D). Adiponectin levels did not change after 6 months (9.54 ± 5.29 μg/mL; *p* = 0.405) but significantly decreased after one year (8.85 ± 4.53 μg/mL; *p* = 0.013) compared to baseline (9.74 ± 4.75 μg/mL) (Figure 1E). Leptin/adiponectin ratios did not change over time (data not shown). Finally, chemerin levels significantly decreased both after 6 months (82.6 ± 20.6 ng/mL; *p* < 0.001) and 12 months (86.7 ± 19.9 ng/mL; *p* < 0.001) versus baseline (111.3 ± 34.7 ng/mL) (Figure 1F).

Lipid fractions, such as TC, TG, HDL-C, and LDL-C, as well as lipid indices (TC/HDL-C and LDL-C/HDL-C) did not change between baseline and 12 months upon anti-TNF therapy (data not shown).

### 3.3. Associations of Metabolic Biomarkers with Disease Activity, Vascular Pathophysiology, and Other Parameters

In the simple correlation analysis, several correlations were found between metabolic and other parameters (data not shown). In general, lipids and lipid ratios variably correlated with age, disease duration, disease activity, CRP, IMT, and PWV (*p* < 0.05). Interestingly, IMT showed negative correlations with LDL and lipid ratios. PON and ARE activity, as well as MPO showed variable associations with age, disease duration, CRP, IMT, and PWV (*p* < 0.05). PON and ARE exerted positive correlations with FMD, but negative associations with disease duration and activity, CRP, IMT, and PWV. MPO showed positive correlations with CRP, IMT, and PWV. Among adipokines, adiponectin correlated with age and PWV, leptin with age, disease activity, CRP, IMT, and PWV, leptin/adiponectin ratios with CRP and PWV, while chemerin only with CRP (*p* < 0.05) (data not shown).

When the metabolic markers were correlated with categorical (binary) variables, obesity was associated with lower HDL-C, higher TC/HDL-C ratio, higher leptin, lower adiponectin levels, and higher leptin/adiponectin ratio (*p* < 0.05) (data not shown). Positive CV history was associated with lower ARE and PON activities, as well as higher leptin levels (*p* < 0.05) (data not shown).

Univariable and multivariable regression analyses were performed in order to determine independent metabolic determinants of IMT, PWV, and FMD (Table 2A), as well as independent determinants of the various metabolic parameters (Table 2B). In the univariable analysis, IMT at various time points positively correlated with TG and leptin and negatively with MPO (*p* < 0.05). PWV was variably, positively associated with TC, LDL, TG, leptin, and leptin/adiponectin ratio and inversely with ARE activity (*p* < 0.05). FMD inversely correlated with TG (*p* < 0.05) (Table 2A). The multivariable analysis confirmed the above associations of IMT with TG, MPO, and leptin and that of PWV with TC, ARE activity, and leptin (*p* < 0.05) (Table 2A).

Similarly, in the univariable analysis, among lipids, TC, and LDL-C were associated with PWV. HDL-C positively correlated with age, CV history, disease duration, and IMT, while inversely with CRP and obesity. TG at baseline negatively correlated with disease activity, but after treatment, it was positively associated with CRP. TG also inversely correlated with FMD (*p* < 0.05). PON activity negatively correlated with age, CV history and IMT, while ARE activity was inversely associated with age, CV history, and PWV (*p* < 0.05). Among adipokines, leptin positively correlated with age, CV history, obesity, disease activity, CRP, IMT, and PWV. Adiponectin was positively associated with age and negatively with obesity. The leptin/adiponectin ratio correlated with obesity and PWV. Finally, chemerin was only associated with CRP (*p* < 0.05) (Table 2B). Among these associations, multivariable analysis confirmed those of HDL-C with age, obesity, disease duration and CRP. LDL correlated with PWV, TG with disease activity, PON activity with age, ARE activity with age and CV history, leptin with obesity and IMT. Adiponectin and leptin/adiponectin ratio also correlated with obesity (*p* < 0.05) (Table 2B).

Finally, RM-ANOVA analysis was performed in order to look for combined determinants of metabolic biomarker changes between baseline and 12 months (Table 3). TC change overtime was associated with treatment and increased baseline leptin levels (*p* = 0.039). HDL changes correlated with treatment and lower baseline CRP (*p* = 0.016). TG changes were associated with treatment and higher adiponectin levels at baseline (*p* = 0.014). Changes in ARE activity correlated with treatment and lower baseline disease activity (*p* = 0.046). Finally, IMT changes were associated with treatment and baseline chemerin (*p* = 0.003) (Table 3).

## 4. Discussion

In this cohort of RA and AS patients, we longitudinally assessed the effects of one-year anti-TNF therapy on metabolic biomarkers in association with markers of disease activity, inflammation, and vascular pathophysiology. Anti-TNF therapy decreased ARE activity, MPO, adiponectin, and chemerin levels after 12 months, while lipids, PON activity, and leptin remained unchanged. Regression analyses indicated associations of IMT, PWV, and FMD indicating vascular pathophysiology with ARE, MPO, leptin, and lipids. On the other hand, these metabolic parameters were significantly associated with disease duration, CV history, CRP, obesity, PWV, and IMT.

In the very same cohort, ETN or CZP therapy significantly improved DAS28 and BASDAI and significantly decreased CRP after 12 months compared to baseline [47]. With respect to vascular pathophysiology, in the same cohort, IMT remained unchanged, while FMD improved and PWV decreased during the one-year anti-TNF treatment period [47]. Regarding full blood counts, hgb, htc, WBC, RBC, PLT, neutrophil, and lymphocyte counts did not change overtime (data not published).

Metabolic syndrome has been associated with arthritides [5,7,8,9,55]. Among metabolic pathways, PON and ARE exert antioxidant and atheroprotective effects [21,22] while MPO has been implicated in both inflammation and CVD [32,33,34]. These physiological and pathophysiological effects may also be present in inflammatory rheumatic diseases, such as RA and AS. In this study, TNF-α inhibition resulted in increased ARE activity, as well as MPO and adiponectin levels after 12 months and increased chemerin levels after 6 and 12 months. At the same time, in this study, TNF inhibition did not alter lipid levels (TC, HDL-C, LDL-C), PON activity and leptin levels. This was accompanied by clinical efficacy, improved FMD, decreased PWV, and unchanged IMT overtime in the very same cohort as already published before [47].

PON and ARE activities are impaired in arthritides. PON1 may also have anti-inflammatory effects [23,24,25,26,27,28]. PON has been implicated in AS, as well [26]. We found variable, inverse correlations of PON and ARE activity with disease duration, disease activity, and CRP. There has been one study showing inverse correlation between PON and disease activity [28]; however, our present study had longer duration and we also included AS patients. In our study, PON activity also correlated with FMD and inversely with positive CV history and IMT, while ARE activity inversely with CV history and PWV. Change of ARE activity overtime was determined by one-year treatment and lower baseline disease activity. These results underscore that PON1 is indeed involved in the maintenance of vascular physiology, acts against atherosclerosis and is inversely regulated by inflammation in arthritides. This is supported by previous studies showing correlations between PON and IMT in AS [26]. As discussed above, ARE activity increased upon anti-TNF therapy. There have been no reports on the possible effects of biologics on ARE. In our study, PON activity did not change upon treatment. However, PON activity is impaired in RA [23] so in our study, TNF inhibition was able to maintain PON activity overtime.

In this study, MPO variably and positively correlated with CRP, IMT and PWV supporting its role in RA-related inflammation and atherosclerosis [35,36]. MPO correlated with diseases activity in other RA studies [56]. In the general population, MPO levels and PON activity were inversely correlated [34], but it was not found under inflammatory conditions in our arthritis patients. We did not find associations between MPO and atherosclerosis. In other studies performed in non-inflammatory diseases, MPO was associated with IMT in metabolic syndrome [57] but not in type 2 diabetes mellitus [58]. In our study, anti-TNF treatment decreased MPO levels after 12 months. We have not found any similar studies on the effects of biologics on MPO.

Among adipokines, leptin and chemerin are clearly pro-inflammatory and pro-atherogenic both in inflammatory and non-inflammatory conditions [5,6,39,42,43,59]. In the present study, leptin showed associations with disease activity, CRP, CV history, obesity, IMT, and PWV indicating that leptin indeed forms a bridge between inflammation and atherosclerosis. Others also found that elevated leptin levels correlated with more severe RA, as well as obesity and CVD in RA [5,39,59]. Leptin/adiponectin ratios correlated with arterial stiffness and obesity. Indeed, this ratio has been associated with atherosclerosis [55] and it seems that it may be a good marker of arterial stiffening. In this study, leptin levels remained unchanged, while adiponectin levels decreased after TNF inhibition. In previous studies, the effects of biologics on leptin and adiponectin levels were highly controversial [5,6,14,39,46]. In the present study, chemerin levels correlated only with CRP, which supports that this is a pro-inflammatory adipokine. In addition, treatment and baseline chemerin levels determined one-year changes in IMT indicating the role of chemerin in atherosclerosis associated with arthritides. Anti-TNF therapy decreased chemerin levels. In other studies, biologics also inhibited chemerin production [5,44,45].

Regarding lipids, the “lipid paradox”, where lipid levels inversely correlate with systemic inflammation, has been identified in inflammatory RMDs [5,16,17,18]. The favorable effects of targeted therapies, mainly IL-6 and JAK inhibitors, are reflected in transient increases in the levels of lipids including TC, LDL-C, HDL-C, and TG [5,6,16,17,19,20]. TNF-α blockers may also, transiently, increase lipid levels but to a lesser extent than IL-6 or JAK inhibitors [5,6,17,18,19]. In our study, none of the lipids changed upon anti-TNF therapy. There have been other studies, where lipids remained unchanged after TNF inhibition [18,60]. In our study, lipids variably and positively correlated with PWV and CV history and inversely with FMD indicating its role in vascular pathophysiology. Lipids and CRP or disease activity inversely correlated with each other at baseline reflecting the lipid paradox. On the other hand, we found positive associations between these parameters after anti-TNF treatment suggesting, that biological therapy indeed dampened inflammation and after one year the lipid paradox may not be present. Finally, treatment together with leptin levels determined changes of TC, while treatment and CRP were negatively associated with that of HDL-C during one-year treatment.

Our study may certainly have some limitations. The relatively small study sample may have obscured potentially significant results. In addition, patients with potentially positive history of CV disease were also included. RA and AS patients were not analyzed separately due to the relatively small number of patients.

## 5. Conclusions

In conclusion, one-year anti-TNF treatment significantly improved ARE activity, MPO levels and chemerin production. Moreover, we found numerous significant, variable associations between lipid, other metabolic markers (PON, ARE, MPO, and adipokines) and vascular pathophysiology. Various studies have been performed on the effects of biologics on lipids, leptin, adiponectin, and imaging markers of atherosclerosis. However, to our knowledge, this may be the first study assessing these parameters together with disease activity, CRP, PON1 activities, MPO, and chemerin. Assessment of various metabolic parameters together with disease activity, markers of inflammation, and ultrasound-based, non-invasive techniques may exert additional value in determining CV burden and in monitoring the effects of biologics on preclinical vascular pathophysiology in relation to clinical efficacy.

## Figures and Tables

**Table 1:** Patient Characteristics.

Characteristic Parameter	Rheumatoid Arthritis	Ankylosing Spindylitis	Total
n	36	17	53
female:male	31:5	3:14	34:19
age (mean ± SD) (range), years	55.9 ± 9.8 (35–83)	43.6 ± 12.4 (24–72)	52.0 ± 12.1 (24–83)
disease duration (mean ± SD) (range), years	9.1 ± 8.3 (1–44)	7.2 ± 7.0 (1–26)	8.5 ± 7.9 (1–44)
age at diagnosis	47.0 ± 8.7 (28–62)	36.4 ± 11.6 (23–50)	43.5 ± 12.1 (23–62)
smoking (current)	7	7	14
positive history of cardiovascular disease	8	1	9
BMI (mean ± SD), kg/m^2^	29.3 ± 3.6	31.1 ± 3.8	29.9 ± 3.7
obesity (BMI > 30 kg/m^2^)	17	11	28
diabetes mellitus history	3	1	4
hypertension history	17	4	21
baseline plasma total cholesterol (mmol/L)	5.65 ± 1.92	5.25 ± 1.34	5.52 ± 1.78
baseline plasma LDL-cholesterol (mmol/L)	3.49 ± 0.92	3.37 ± 0.66	3.45 ± 0.87
baseline plasma HDL-cholesterol (mmol/L)	1.58 ± 0.39	1.40 ± 0.22	1.52 ± 0.34
baseline plasma triglyceride (mmol/L)	1.62 ± 0.55	1.47 ± 0.36	1.57 ± 0.41
rheumatoid factor positivity, n (%)	26 (72)	-	-
ACPA positivity, n (%)	21 (58)	-	-
DAS28 (baseline) (mean ± SEM)	5.00 ± 0.86	-	-
BASDAI (baseline) (mean ± SEM)	-	5.79 ± 1.19	-
Treatment (etanercept, certolizumab pegol)	20 ETN, 16 CZP	17 ETN	37 ETN, 16 CZP

Abbreviations: ACPA, anti-citrullinated protein antibodies; BASDAI, Bath ankylosing spondylitis disease activity index; BMI, body mass index; DAS28, 28-joint disease activity score; SD, standard deviation.

**Table 2:** Univariable and Multivariable Regression Analysis of Metabolic and Vascular Associations *.

**A.** Determination of vascular pathophysiology by metabolic markers									
**Dependent Variable**	**Independent Variable**	**Univariable Analysis**				**Multivariable Analysis**			
		**β**	***p***	**B**	**CI 95%**	**β**	***p***	**B**	**CI 95%**
IMT-0	Leptin-0	0.372	0.015	0.001	0–0.002				
	Leptin-6	0.361	0.019	0.001	0–0.002				
	Leptin-12	0.381	0.013	0.001	0–0.002				
IMT-6	Triglyceride-0	0.348	0.018	0.062	0.011–0.113	0.342	0.012	0.064	0.015–0.114
	Myeloperoxidase-0	−0.378	0.001	0		−0.382	0.006	0	
	Myeloperoxidase-6	−0.294	0.042	0					
PWV-0	Total cholesterol-0	0.436	0.004	0.763	0.266–1.3	0.436	0.004	0.763	0.266–1.3
	LDL-cholesterol-0	0.360	0.019	0.665	0.115–1.216				
	Arylesterase-0	−0.338	0.030	−0.011	−0.021 to −0.001				
PWV-6	Arylesterase-0	−0.359	0.014	−0.010	−0.018 to −0.002	−0.255	0.005	−0.007	−0.014–0
	Arylesterase-6	−0.301	0.042	−0.015	−0.029 to −0.001				
	Leptin-0	0.435	0.002	0.020	0.007–0.032				
	Leptin-6	0.545	<0.001	0.024	0.013–0.035	0.490	<0.001	0.021	0.010–0.030
	Leptin-12	0.519	<0.001	0.024	0.012–0.035				
	Leptin/Adiponectin-6	0.968	0.011	0.121	0.029–0.212				
	Leptin/Adiponectin-12	0.312	0.033	0.089	0.008–0.170				
PWV-12	Triglyceride-0	0.344	0.017	1.289	0.246–2.331				
FMD-6	Triglyceride-6	−0.291	0.040	−2.214	−4.424 to −0.004				
**B.** Determination of metabolic biomarkers by disease activity and other parameters									
**Dependent** **Variable**	**Independent Variable**	**Univariable Analysis**				**Multivariable Analysis**			
		**β**	***p***	**B**	**CI 95%**	**β**	***p***	**B**	**CI 95%**
Total cholesterol-0	PWV-0	0.436	0.004	0.242	0.082–0.402				
Total cholesterol-6	PWV-0	0.471	0.002	0.324	0.130–0.518				
Total cholesterol-12	PWV-0	0.339	0.028	0.190	0.021–0.358				
HDL-cholesterol-0	age	0.291	0.035	0.010	0.001–0.020	0.289	0.022	0.010	0.001–0.018
	CRP-0	−0.319	0.020	−0.008	−0.015 to −0.001	−0.404	0.001	−0.010	−0.016 to −0.004
	Cardiovascular history	0.320	0.024	0.272	0.038–0.506				
	obesity	−0.413	0.003	−0.343	−0.563 to −0.123	−0.319	0.003	−0.319	−0.521 to −0.118
HDL-cholesterol-6	age	0.317	0.021	0.012	0.002–0.023				
	disease duration	0.437	0.001	0.026	0.011–0.041	0.437	0.001	0.026	0.011–0.041
	IMT-0	0.335	0.030	1.886	0.189–3.583				
	Cardiovascular history	0.338	0.016	0.318	0.061–0.575				
	obesity	−0.399	0.004	−0.367	−0.612 to −0.122				
HDL-cholesterol-12	age	0.437	0.001	0.018	0.008–0.028	0.370	0.006	0.015	0.004–0.025
	disease duration	0.377	0.005	0.024	0.007–0.040				
	obesity	−0.369	0.008	−0.361	−0.625 to −0.097	−0.281	0.034	−0.275	−0.528 to −0.021
	Cardiovascular history	0.341	0.015	0.331	0.066–0.596				
LDL-cholesterol-0	PWV-0	0.360	0.019	0.195	0.034–0.357				
LDL—cholesterol-6	PWV-0	0.468	0.002	0.271	0.108–0.435				
LDL-cholesterol-12	PWV-0	0.426	0.005	0.213	0.069–0.358				
Triglyceride-0	DAS/BASDAI-0	−0.403	0.005	−0.211	−0.355 to −0.067				
Triglyceride-6	FMD-6	−0.291	0.040	−0.038	−0.077–0				
Triglyceride-12	CRP-6	0.742	<0.001	0.105	0.078–0.132				
	CRP-12	0.389	0.004	0.069	0.023–0.115				
Paraoxonase-0	age	−0.317	0.021	−2.082	−3.835 to −0.329				
Paraoxonase-6	age	−0.386	0.004	−2.736	−4.676 to −0.916	−0.386	0.004	−2.736	−4.676 to −0.916
	IMT-0	−0.320	0.039	−0.063	−0.121 to −0.004				
	Cardiovascular history	−0.301	0.034	−53.999	−103.686 to −4.312				
Paraoxonase-12	age	−0.383	0.005	−2.626	−4.404 to −0.848	−0.383	0.005	−2.626	−4.404 to −0.848
	Cardiovascular history	−0.276	0.050	−46.751	–93.96–0.459				
Arylesterase-0	age	−0.372	0.007	−1.768	−3.020 to −0.516	−0.372	0.007	−1.768	−3.020 to −0.516
	PWV	−0.338	0.030	−10.126	−19.248 to −1.005				
	Cardiovascular history	−0.322	0.024	−37.454	−69.749 to −5.160				
Arylesterase-6	age	−0.376	0.006	−1.092	−1.860 to −0.323	−0.376	0.006	−1.092	−1.860 to −0.323
	PWV-6	−0.301	0.042	−6.180	−12.138 to −0.222				
	Cardiovascular history	−0.353	0.013	−24.614	−43.729 to −5.499				
Arylesterase-12	Cardiovascular history	−0.297	0.038	−16.903	−32.840 to −0.967				
Leptin-0	age	0.276	0.045	0.887	0.019–1.754				
	IMT-0	0.372	0.015	160.2	32.464–287.8	0.378	0.009	162.8	42.652–283.0
	obesity	0.375	0.007	29.612	8.380–50.844	0.345	0.017	25.668	4.914–46.422
	Cardiovascular history	0.280	0.049	21.946	0.116–43.776				
Leptin-6	DAS/BASDAI-6	0.322	0.020	11.664	1.932–21.396				
	IMT-0	0.361	0.019	156.9	27.293–286.6	0.282	<0.001	122.843	22.469–223.216
	PWV-6	0.545	<0.001	12.342	6.648–18.036	0.452	<0.001	10.152	4.946–15.358
	obesity	0.455	0.001	34.121	14.733–53.508	0.405	0.001	30.407	13.314–47.499
	Cardiovascular history	0.317	0.025	23.594	3.095–44.094				
Leptin-12	DAS/BASDAI-12	0.277	0.006	13.124	3.972–22.276				
	CRP-12	0.369	0.006	1.745	0.511–2.979				
	IMT-0	0.381	0.013	162.6	36.488–288.8	0.307	0.003	27.713	10.345–45.081
	PWV-6	0.519	<0.001	11.372	5.752–16.992	0.425	0.001	9.373	4.083–14.662
	obesity	0.427	0.002	31.163	12.026–50.300	0.376	0.003	27.713	10.345–45.081
	Cardiovascular history	0.315	0.016	22.780	2.834–42.727				
Adiponectin-0	age	0.286	0.038	0.113	0.007–0.219				
	obesity	−0.519	<0.001	−4.887	−7.223 to −2.552	−0.519	<0.001	−4.887	−7.223 to −2.552
Adiponectin-6	obesity	−0.497	<0.001	−5.241	−7.899 to −2.583				
Adiponectin-12	age	0.348	0.011	0.134	0.0320.235				
	obesity	−0.495	<0.001	−4.573	−6.900 to −2.246	−0.495	<0.001	−4.573	−6.900 to −2.246
Leptin/Adiponectin-6	PWV-6	0.368	0.011	1.121	0.270–1.972				
	Obesity	0.509	<0.001	5.286	2.689–7.884	0.509	<0.001	5.286	2.689–7.884
Leptin/Adiponectin-12	PWV-6	0.312	0.033	1.095	0.094–2.096				
	Obesity	0.046	0.001	5.210	2.293–8.128	0.046	0.001	5.210	2.293–8.128
Chemerin-0	CRP-0	0.378	0.005	0.767	0.239–1.295				
Chemerin-6	CRP-0	0.320	0.020	0.385	0.064–0.706				
	CRP-6	0.523	<0.001	1.131	0.612–1.649				

* Only significant correlations are listed in this table. Abbreviations: BASDAI, Bath ankylosing spondylitis disease activity index; CRP, C-reactive protein; DAS, disease activity score; FMD, flow-mediated vasodilation; HDL, high-density lipoprotein; IMT, intima-media thickness; LDL, low-density lipoprotein; PWV, pulse-wave velocity; the numbers -0, -6, and -12 indicate values at baseline and after 6 and 12 months of treatment.

**Table 3:** Significant results of general linear model repeated measures analysis of variance (RM-ANOVA) test determining the effects of treatment and other independent variables on metabolic parameters as dependent variables *.

Dependent Variable	Effect	F	*p*	Partial η^2^
Total cholesterol 0-12	Treatment * Leptin-0	4.475	0.039	0.081
HDL-cholesterol 0-12	Treatment * CRP-0 (inv)	4.499	0.016	0.153
Triglyceride 0-12	Treatment * Leptin/Adiponectin-0	6.469	0.014	0.115
Arylesterase 0-12	Treatment * DAS28/BASDAI-0 (inv)	3.315	0.046	0.131
IMT 0-12	Treatment * Chemerin-0	6.933	0.003	0.262

* Only significant correlations are listed in this table. Abbreviations: BASDAI, Bath ankylosing spondylitis disease activity index; CRP, C-reactive protein; DAS, disease activity score; HDL, high density lipoprotein; IMT, intima-media thickness; (inv), inverse association; RM-ANOVA; repeated measures analysis of variance. The numbers -0 and -12 indicate values at baseline and after 12 months of treatment.

## Data Availability

Data are available from the authors upon reasonable request.

## Abbreviations

ACPA, anti-citrullinated protein antibody; ARE, arylesterase; AS, ankylosing spondylitis; BASDAI, Bath Ankylosing Spondylitis Disease Activity Index; CCP, cyclic citrullinated peptide; CI, confidence interval; CRP, C-reactive protein; CV, cardiovascular; CVD, cardiovascular disease; CZP, certolizumab pegol; DAS, disease activity index; ETN, etanercept; FMD, flow-mediated vasodilation; HDL-C, high density lipoprotein cholesterol; hs, high sensitivity; IL, interleukin; IMT, intima-media thickness; JAK, Janus kinase; LDA, low disease activity; LDL-C, low density lipoprotein cholesterol; Lp(a), lipoprotein (a); MACE, major cardiovascular event; MPO, myeloperoxidase; PON, paraoxonase; PWV, pulse-wave velocity; RA, rheumatoid arthritis; RF, rheumatoid factor; RM-ANOVA, repeated measures analysis of variance; RMD, rheumatic and musculoskeletal disease; SC, subcutaneous; TC, total cholesterol; TG, triglyceride; TNF, tumor necrosis factor.
